# Effects of anesthetics on development of gynecological cancer

**DOI:** 10.3389/fcell.2025.1587548

**Published:** 2025-04-16

**Authors:** Yingxiang Cheng, Yunzhi Wu, Lingzhong Xu

**Affiliations:** ^1^ Department of Anesthesiology, Zhenjiang Fourth People’s Hospital, Zhenjiang, China; ^2^ Department of Thoracic Surgery, Affiliated People’s Hospital of Jiangsu University, Zhenjiang, China

**Keywords:** cervical cancer, ovarian cancer, endometrial cancer, intravenous anesthetics, volatile anesthetics, local anesthetics, opioids, dexamethasone

## Abstract

Gynecological cancers remain a leading cause of cancer among female patients, and surgery continues to be the primary therapeutic approach. Anesthesia is an indispensable component of perioperative period. In recent years, the influence of anesthesia drugs on cancer has become one of the focuses of anesthesiologists. Anesthetic drugs may influence cancer metabolic reprogramming and modulate immune function through the hypothalamic-pituitary-adrenal (HPA) axis and the sympathetic nervous system (SNS). Emerging evidence suggests that the choice of anesthetic agents could affect the prognosis of gynecological cancers. This review explores the relationship between anesthetic drugs and gynecological cancers (cervical cancer, ovarian cancer, and endometrial cancer), elucidating their effects on cancer prognosis through cellular pathways, metabolic regulation, and immune mechanisms. The findings aim to guide clinical decision-making and evaluate optimal perioperative anesthetic management strategies for gynecological cancer patients.

## 1 Introduction

Cancer is a major contributor to the global burden of disease. Global statistics for 2022 indicated that there were nearly 20 million new cancer cases and nearly 10 million cancer deaths, and the number of new cancer cases was expected to increase by 77% by 2050 (Bray et al., 2024). Gynecologic cancers were the second most common cause of cancer in women. Cervical cancer ranked fourth and ovarian cancer sixth among women’s cancers globally. Analysis of global epidemiologic data showed that women in China had a higher likelihood of developing cervical cancer, while women in the United States and the United Kingdom exhibited higher incidence rates of uterine corpus tumors (Diao et al., 2024). The mean age at diagnosis for cervical cancer was 53 years, while the mean age at death from the disease was 59 years (Arbyn et al., 2020). Ovarian cancer occurred predominantly in postmenopausal women, with higher rates in European and North American countries (Cabasag et al., 2020).

With the popularization of HPV vaccine and early surgical treatment, the survival rate and quality of life of patients with gynecological cancer have been significantly improved. However, postoperative recurrence and metastasis are the common concerns of doctors and patients, which bring great psychological pressure and economic burden to patients. The progression of cancer is closely related to the perioperative period, which can promote angiogenesis, the shedding of circulating tumor cells (CTCs) and suppress immunity. Anesthesia is an important part of the perioperative period. A lot of studies have found that anesthetics and techniques have a certain impact on the progression of cancer, but the effects of different anesthetic techniques and drugs are inconsistent. There is a notable paucity of comprehensive reviews addressing the relationship between anesthesia and gynecological cancer. In this review, we first delineate the molecular mechanisms underlying cancer metastasis, followed by a systematic synthesis of existing evidence on the impacts of anesthetics on gynecological cancers and their associated pathophysiological pathways. This synthesis not only provides novel directions for future research in gynecological oncology but also establishes a strengthened theoretical foundation for exploring therapeutic targets, enhancing prognostic outcomes, and improving quality of life in affected patients.

## 2 Mechanisms of tumor metastasis

Metastasis, a hallmark feature of cancer, constitutes the primary cause of mortality in most cancer patients. Therefore, inhibiting cancer metastasis is critical for improving survival rate (Park et al., 2022). Direct therapeutic targeting of metastasis in cancer patients may enhance progression-free survival (PFS), representing a promising treatment strategy (Miszczyk et al., 2024). Therefore, understanding the mechanisms of cancer metastasis is essential.

**FIGURE 1:**
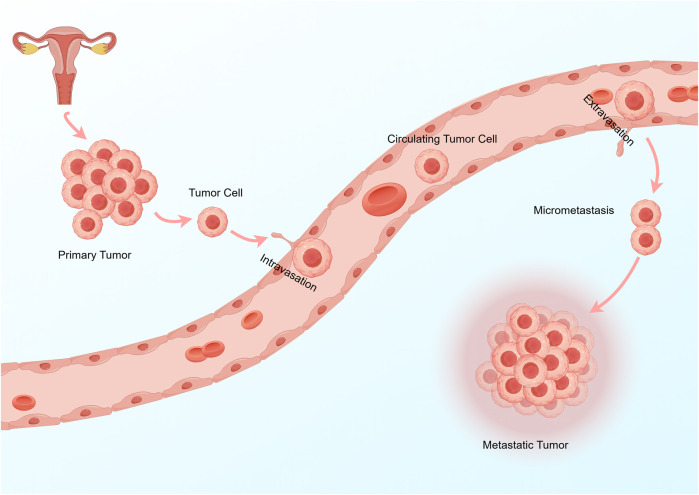
The key steps of tumor cell metastasis are: 1 Tumor cells reach the surrounding blood vessels or lymphatic vessels. 2 Tumor cells become CTCs after intravasation. 3 Survival of CTCs in the circulation or lymphatic system. 4 CTCs extravasation and metastasis to distant organs.

The metastasis of tumor cells mainly goes through the following steps: 1. Epithelial-mesenchymal transition (EMT): This is necessary for metastasis, which makes residual tumor cells acquire motility, invasion and exudation. 2. Intravasation: Tumor cells become circulating tumor cells (CTCs) after intravasation, which is the key rate-limiting step and determines the number of CTCs in the circulation. 3. Circulation: Only a small number of CTCs survive in the circulatory system, which is affected by various factors, such as immune cell and platelet activity. 4. Colonization: CTCs can only be colonized by metastasis into the microvasculature of distant organs and extravasation (Sulekha Suresh and Guruvayoorappan, 2023). This can be simply understood as tumor cells intravasate into the blood or lymphatic vessels and then spread. After reaching the metastatic site, CTCs extravasate to the designated organ and survive and form metastatic tumors (Kitamura et al., 2015; Figure 1). The interaction between tumor metastasis and tumor metabolism affects the metastasis and progression of tumors.

Metabolic reprogramming, a hallmark of malignant tumors, facilitates accelerated cellular growth and proliferation through the modulation of energy metabolism. Under certain conditions, metabolic reprogramming can be used to diagnose, treat cancer, and monitor. In cultured cancer cells, typical cancer cell metabolic activities include aerobic glycolysis, glutamine catabolism, and macromolecular synthesis (Faubert et al., 2020). Cancer metabolism drives EMT, which in turn can reprogram the cancer metabolism. Abnormal tumor metabolism, especially the enhanced aerobic glycolysis, can increase the acid load of tumor cells while providing energy for tumor cells. Thus, cancer metabolism is involved in the growth and invasion of malignant tumors (Huang and Zong, 2017). Angiogenesis affects the intravasation of tumor cells, and tumor cells and the surrounding stroma secrete vascular endothelial growth factor (VEGF), causing neovascularization. HIF-1α is positively correlated with VEGF, and HIF-1α may promote tumor angiogenesis and tumor cell metastasis by up-regulating VEGF (Apte et al., 2019; Tian et al., 2018). The circulation and colonization of tumor cells are related to many factors, among which immune factors play an important role. Metabolites produced by tumor cells affect the generation and function of immune cells in the tumor microenvironment (TME). For example, acidified TME can inhibit the function of immune cells and eventually lead to immune escape (Xia et al., 2021). However, different metabolites of tumor cells have different effects on immune cells, so more researches are needed to explore, but the intervention of tumor metabolism is worth looking forward to in cancer immunotherapy.

**FIGURE 2:**
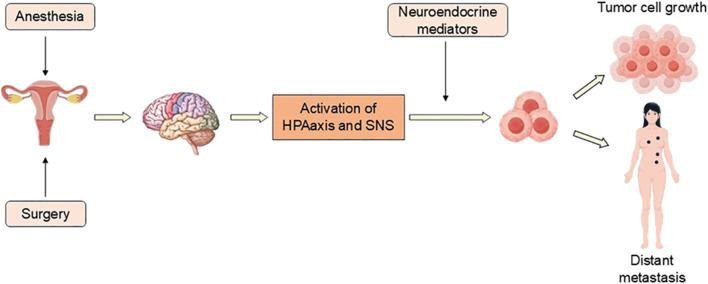
Surgery and anesthesia stimulate the activation of HPA axis and SNS, and release a series of neuroendocrine mediators, which affect tumor cells and their microenvironment, resulting in tumor recurrence or distant metastasis.

Surgical treatment can remove tumor tissue, but it also provides the opportunity for residual tumor cells to intravasate into systemic circulation and lymphatic channels, thereby potentially initiating metastatic dissemination (Neeman and Ben-Eliyahu, 2013). However, not all tumor cells can metastasize, and generally only a few tumor cells can metastasize, which indicates that the metastasis of tumor cells is relatively complex and there is a “survival of the fittest”. This situation demonstrates essential interconnectivity with the human innate immune system and adaptive immune system. The innate immune system serves as the primary defensive barrier against cancer progression and orchestrates the activation of adaptive immune responses. The innate immune system includes immune cells, cytokines, chemokines and proteins of the complement system, which can activate the adaptive immune system through the complement system, promoting immune cell-mediated lysis and phagocytosis of tumor cells (Maiorino et al., 2022). NK cells are a major component of innate immunity, which can kill cells, promote the production of proinflammatory cytokines, and enhance the cytotoxicity and persistence of NK cells *in vivo*. NK cells are one of the targeted therapies for cancer and play an important role in tumor defense (Myers and Miller, 2021). Tumor-associated macrophages (TAMs) represent a phenotypically diverse and highly plastic cell population in TME. TAMs can induce EMT of tumor cells, alter the extracellular matrix (ECM), participate in the vascular signaling cascade, and enhance tumor cell migration, permeability, and spread (Friedman-DeLuca et al., 2024; Figure 2).

The immune system is regulated by the hypothalamic-pituitary-adrenal (HPA) axis and the sympathetic nervous system (SNS), in which glucocorticoids and catecholamines can inhibit immune function. The HPA axis and SNS can activate various complex cellular pathways in tumors to promote tumor growth and progression (Antoni et al., 2006). Both surgery and anesthesia can activate the SNS and HPA axis. Surgery can activate the stress response, promote the release of angiogenic factors, inhibit NK cells and cell-mediated immunity to promote tumor metastasis. Anesthesia can lead to immunosuppression and accelerate cancer recurrence (Kim, 2018).

## 3 Anesthetics and gynecologic cancer outcomes

**FIGURE 3:**
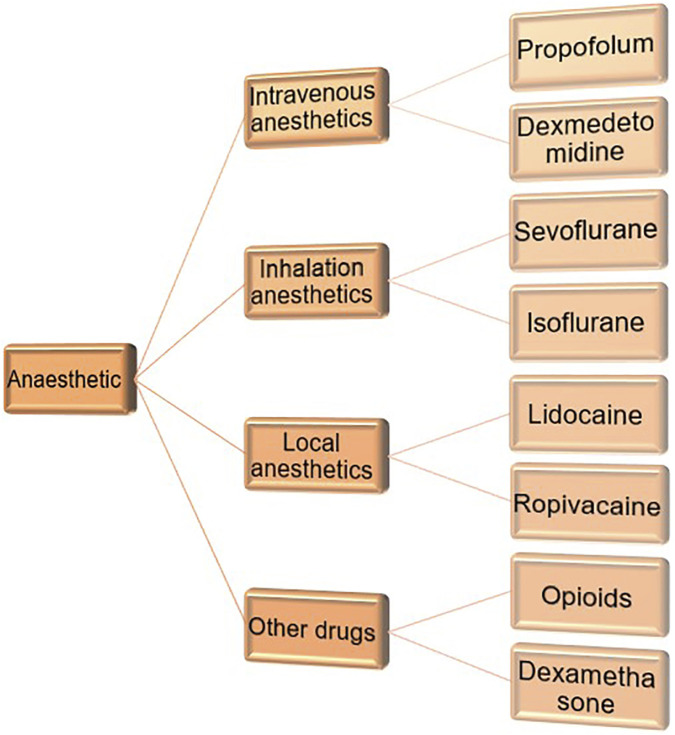
Classification of commonly used anesthetics in clinical practice.

In order to obtain safer and more effective anesthetic regimens, reduce cancer recurrence and prolong postoperative survival, we need to further understand the relationship between anesthetic drugs and the prognosis of gynecological tumors. The classification of anesthetics is briefly introduced (Figure 3).

### 3.1 Intravenous anesthetics

#### 3.1.1 Propofol

Propofol, an intravenous hypnotic drug, stands as one of the most extensively utilized intravenous anesthetics in surgery. It works by enhancing the inhibitory neurotransmitter γ-aminobutyric acid (GABA) on GABA_A_ receptor. In recent years, propofol has been found to be associated with various types of cancer, mainly manifested as anti-tumor effects and minor pro-tumor effects (Farooqi et al., 2020; Sahinovic et al., 2018; Xu Y. et al., 2020). In addition, propofol can regulate postoperative immune function. Although it cannot directly reverse the immune changes caused by surgery and the activation of HPA and SNS, propofol can have effects on different immune cells, which may be the potential mechanism of propofol anti-tumor (Gu et al., 2022).

Propofol can not only affect cancer through immune function, but also affect the metabolic reprogramming of tumor cells, glycolysis is one of the main ways. Glycolysis is the predominant energy source for tumor cells (aerobic or anaerobic), also known as the Warburg effect. Therefore, targeting the glycolytic pathway of tumor cells has been a research hotspot in recent years (Qu et al., 2022; Wu Z. et al., 2022; Zhao et al., 2020).

##### 3.1.1.1 Cervical cancer

Propofol can reduce the viability of cervical cancer cells and block the fusion of autophagosomes and lysosomes. At the same time, propofol can induce ER stress and destroy intracellular Ca2^+^ balance, and enhance the accumulation of autophagosomes. Propofol exerts anti-tumor effect by impairing autophagic flux (Chen et al., 2018). Propofol and paclitaxel can induce mitochondrial morphological changes related to apoptosis of cervical cancer cells and enhance intracellular ROS expression. In addition, propofol can enhance paclitaxel-induced death of cells, and propofol and paclitaxel have synergistic anticancer effects on cervical cancer cells (Zhao et al., 2022).

In addition to the anti-cancer effect of propofol on cervical cancer cells by regulating the immune response, it can also reduce the migration ability of cells by changing the ultrastructure of the cell surface, inhibit the proliferation of cells, and promote cell apoptosis (Zhang F. et al., 2016). MIR155HG was shown to be significantly upregulated in cervical cancer patients, and propofol reduced the expression of MIR155HG, thereby inhibiting the growth and invasion of cervical cancer cells, and this effect was confirmed both *in vivo* and *in vitro* (Du et al., 2021). Stathmin protein is highly expressed in cervical cancer tissues and is related to clinical stage, T classification and metastasis, which is a valuable prognostic marker for cervical cancer patients (Xi et al., 2009). Studies have found that high concentrations of propofol enhance the cytotoxicity of paclitaxel against cervical cancer cells by down-regulating stathmin 1 expression, but clinical concentrations of propofol may not have this effect (Jin et al., 2023). However, clinically relevant concentrations of propofol can effectively inhibit the invasion of human cervical cancer cells by regulating Rho A (Mammoto et al., 2002). There are many reasons for these different results, such as different formulations of propofol, different manufacturers, and different experimental conditions, which can lead to different results. Therefore, we still need to continue to explore the specific mechanisms and signaling pathways of propofol against cancer.

Propofol can suppress cervical cancer cell viability, and enhance cisplatin-mediated apoptosis by inhibiting the EGFR/JAK2/STAT3 pathway, thereby enhancing the anti-tumor effect of cisplatin (Li et al., 2017). Propofol can inhibit the HOTAIR-mediated mTOR/p70S6K pathway in cervical cancer, thereby inhibiting tumor enlargement and cell viability, and promoting apoptosis (Zhang et al., 2015). Propofol inhibits cervical cancer cell viability, colony formation, invasion and migration, and promoting apoptosis by regulating the HOTAIR/miR-129-5p/RPL14 pathway (Sun D. et al., 2021). The above studies suggest that although the effects of clinically relevant concentrations of propofol on cervical cancer are still controversial, it has substantial advantages in the treatment of cervical cancer *in vitro* and *in vivo*.

##### 3.1.1.2 Ovarian cancer

Upregulated miR-125a-5p inhibits the glycolysis in cancer (Huang et al., 2018). Propofol can inhibit the proliferation, migration and metastasis of ovarian cancer cells by enhancing miR-125a-5p, which targets LIN28B (Zeng et al., 2020). Whether propofol can affect the glycolysis of ovarian cancer cells by altering miR-125a-5p needs to be explored by more studies. Nuclear factor kappa B (NF-κB) serves as a pivotal mediator in regulating inflammatory responses, including innate and adaptive immunity. Its activation is recognized as an integral component of the stress response and involved in cancer processes (Barnabei et al., 2021). Propofol inhibits the cell viability, migration and invasion, and induces apoptosis, which is related to the upregulation of miR-9 expression and the inhibition of NF-κB and MMP-9 expression in ovarian cancer cells (Huang et al., 2016). In addition to down-regulating NF-κB and MMPs, propofol also exerts anti-tumor effects through other pathways.

Glutaminolysis, another metabolic feature of cancer cells, is one of the significant mechanisms in the development of ovarian cancer and may be a novel therapeutic target for ovarian cancer (Fasoulakis et al., 2023). MiR-145 inhibits the glutaminolysis in ovarian cancer cells (Li et al., 2019). Propofol can inhibit circVPS13C, upregulate miR-145 expression, thereby inhibiting cell viability, cell cycle, migration, proliferation and invasion, and increasing cell apoptosis rate. This is the first time that propofol has been found to work through circVPS13C/miR-145/MEK/ERK signaling in ovarian cancer cells (Lu et al., 2021). The aberrant activation of JAK2/STAT3 signaling has been detected across multiple solid tumors, where it drives tumorigenesis, tumor survival, proliferation, and metastatic. Consequently, the JAK2/STAT3 axis emerges as a rational therapeutic target for cancer (Mengie Ayele et al., 2022). Propofol can inhibit the proliferation, migration, invasion and induce apoptosis of human ovarian cancer cells by down-regulating the expression of HOST2 and inhibiting the JAK2/STAT3 signaling pathway in a dose-dependent manner (Shen et al., 2021).

The resistance of cancer cells to chemotherapy drugs has been a problem faced by patients for a long time. Propofol inhibits the resistance of ovarian cancer cells to cisplatin by regulating the miR-374a/FOXO1 signaling pathway, which provides more favorable evidence for propofol in the anesthesia of ovarian cancer patients (Sun et al., 2020). Propofol has synergistic effects with cisplatin and doxorubicin in ovarian cancer cells, depending on p53 status, and may reduce the required chemotherapy dose and related side effects (Sue et al., 2024). In addition to inhibiting the resistance of ovarian cancer cells to cisplatin, propofol can also enhance the killing effect of paclitaxel on ovarian cancer cells by inhibiting the expression of Slug (Wang et al., 2013).

Although the inhibiting effect of propofol on ovarian cancer cells has been substantiated in most studies, the molecular mechanism of its anti-cancer effect in ovarian cancer is not clear. In addition to the anti-cancer effect of propofol alone on ovarian cancer cells, propofol combined with chemotherapy drugs can also enhance the anti-cancer effect and drug resistance of chemotherapy drugs. Therefore, it is of great significance to explore the effect of propofol in ovarian cancer.

##### 3.1.1.3 Endometrial cancer

Abnormal Wnt signaling can affect glycolysis, glutaminolysis and lipogenesis metabolism of tumor cells, and regulate tumor TME and immune function (El-Sahli et al., 2019). Propofol inhibits Sox4 expression by regulating Wnt/β-catenin signaling pathway, thereby inhibiting the proliferation, migration and invasion of endometrial cancer cells and promoting apoptosis in a dose-dependent manner, which elucidated the molecular mechanism of propofol affecting endometrial cancer (Du et al., 2018). However, there are few studies on the effect of propofol on endometrial cancer, and more studies are needed to explore the effect of propofol on endometrial cancer.

In summary, propofol can inhibit the proliferation, migration and invasion, and promote apoptosis of cervical, ovarian and endometrial cancer cells. However, the mechanism of action of propofol in gynecological tumors still needs to be explored.

#### 3.1.2 Dexmedetomidine (DEX)

Dexmedetomidine (DEX) is an adrenergic receptor agonist with a higher selectivity for α_2_-receptors (α_2_: α_1_ ratio of 1,620:1). It exerts sedative, anxiolytic, sympatholytic and analgesic effects mainly by activating presynaptic and postsynaptic α_2_-receptors in the locus cereus. In 2008, DEX was approved for surgical use in the United States (Weerink et al., 2017). α_2_-receptors are widely found in immune tissues and mediate the biological behavior of the inflammatory immune system. Surgical trauma leads to stress response and aggravates perioperative inflammation and immunosuppression. Perioperative infusion of DEX can inhibit the release of cortisol, norepinephrine, and epinephrine, increase the number of T cells, B cells, and NK cells, and reduce perioperative stress and inflammation (Chen et al., 2022; Wang et al., 2019).

##### 3.1.2.1 Cervical cancer

Th1 cells are the main effector of immune response, which can enhance anti-tumor immunity, while Th2 cells inhibit anti-tumor immunity. There is Th1/Th2 imbalance in cervical cancer patients, and the immune function of the body is weakened, which is related to the occurrence and development of cervical cancer. Balancing the expression of Th1 and Th2 cells can improve the immunosuppression of cervical cancer patients after surgery (Lin et al., 2019; Ma et al., 2015). In addition to immune function, vascular endothelial growth factor (VEGF) is critically implicated in cervical cancer angiogenesis, proliferation, and metastatic progression. VEGF is significantly overexpressed in patients with cervical squamous cell carcinoma, and patients with high VEGF expression have a worse survival rate (Kotowicz et al., 2016; Mitsuhashi et al., 2005; Zhang L. et al., 2016). Most studies estimate Th1/Th2 balance by calculating IFN-γ/IL-4. DEX combined with ketorolac increases IFN-γ concentration and IFN-γ/IL-4 ratio, which improves Th1/Th2 imbalance and improves the immune function of cervical cancer patients after surgery. In addition, DEX combined with ketorolac can reduce serum VEGF level, which may reduce the risk of cervical cancer recurrence (Ao et al., 2024). This is a study of the effect of DEX combined with other drugs on cervical cancer. The effect of DEX alone on cervical cancer is still poorly studied, but it is worth looking forward to the application prospect of DEX in cervical cancer.

##### 3.1.2.2 Ovarian cancer

A retrospective analysis of patients undergoing radical ovarian cancer surgery revealed that, compared to the Midazolam group, the DEX group had stable hemodynamics, reduced the expression of TNF-α and IL-6, and suppressed perioperative stress responses (Liu et al., 2019). Therefore, Dex can reduce the expression of inflammatory factors and regulate immunity, and play a protective role in postoperative metastasis and recurrence of ovarian cancer. DEX can inhibit the surgical stress response and the release of stress mediators, promote the recovery of NK cell activity, and reduce the level of TNF-α, so it can be used as an effective immunomodulatory drug for patients with ovarian cancer during the perioperative period (Shin et al., 2021).

DEX treatment showed that DEX promoted the expression of miR-185, inhibited sex determining region Y-box 9 (SOX9) expression, and inactivated the downstream Wnt/β-catenin signaling pathway to inhibit the proliferation, invasion and migration of ovarian cancer cells (Tian et al., 2021). As mentioned previously, the Wnt/β-catenin signaling pathway affects the metabolism of tumor cells, and DEX can inhibit glycolysis of tumor cells (Zhu and Zhang, 2024). Whether DEX can regulate glycolysis of ovarian cancer through Wnt/β-catenin signaling pathway and affect the progression of ovarian cancer needs to be further explored. Insulin-like growth factor 2 (IGF2) signaling pathway plays a pivotal role in tumorigenesis. IGF2 can enhance the glycolytic pathway and promote the migration, invasion and proliferation of ovarian cancer cells (Ji et al., 2024). DEX can reduce the invasion and migration and enhance the immune function of ovarian cancer cells by inhibiting the IGF2 signaling pathway (H. Tian et al., 2019). The above studies provide a molecular mechanism for DEX to play an anti-cancer role in ovarian cancer, which has a positive significance for the treatment of ovarian cancer.

In conclusion, DEX exhibits predominant anti-cancer effects in cervical and ovarian cancers. Clinical studies indicates that DEX demonstrates protective properties against cancer recurrence in these cancers, so DEX is one of the ideal anesthesia drugs in the perioperative period.

### 3.2 Volatile anesthetics

#### 3.2.1 Sevoflurane

Sevoflurane has different effects on oxidative stress and inflammation in different scenarios. The results of cell and animal experiments have found that sevoflurane has antioxidant and anti-inflammatory effects on a variety of cells except nerve cells. Sevoflurane can promote cancer progression by affecting innate and adaptive immunity, increasing the expression of catecholamines and cytokines, and inhibiting immune function (Choi and Hwang, 2024; Lee et al., 2015; Stollings et al., 2016).

##### 3.2.1.1 Cervical cancer

Laparoscopic radical hysterectomy is a common surgical method for cervical cancer. In the study of these patients, it was found that compared with sevoflurane anesthesia, propofol had less damage to cellular immune function in cervical cancer patients. Compared with propofol group, the ratios of CD3^+^, CD4^+^ T cells, CD4^+^/CD8^+^ and NK cells were lower and the index recovery was later in sevoflurane group (Liu et al., 2016). Compared with propofol, sevoflurane has a greater impact on the immune function of patients with cervical cancer, and may be more unfavorable for the metastasis and recurrence of patients with cervical cancer. In addition, it has been found that sevoflurane can increase the expression of migration-related proteins Ezrin and MMP2 and apoptosis-related protein BCL-2 in cervical cancer cells, but reduce the expression of pro-apoptotic protein BAX, thereby promoting the proliferation and migration of cervical cancer cells and inhibiting the apoptosis of cervical cancer cells, while it has no effect on the sensitivity of cervical cancer cells to cisplatin (Xue et al., 2019).

However, the effects of sevoflurane on cervical cancer are not all negative. A study confirmed that Ras and RhoA are the targets of sevoflurane in the treatment of cervical cancer and found that sevoflurane can inhibit the downstream signaling pathways Ras/Erk/Akt and Rho/MYPT1/MLC of both targets. Sevoflurane plays an anti-migration role by inhibiting RhoA and anti-proliferation role by inhibiting Ras, and can also improve the chemotherapy sensitivity of cervical cancer cells (Ding et al., 2019). There are many reasons for these different conclusions, and there are differences between humans, animals and cells. Moreover, sample size and experimental environment will affect the experimental results. Therefore, more experiments are still needed to explore the relationship between sevoflurane and cervical cancer.

##### 3.2.1.2 Ovarian cancer

Through activation of MAPK signaling, it can cause ER stress and apoptosis in ovarian cancer cells (Li G. N. et al., 2022). Treatment of ovarian cancer cells with three different concentrations of sevoflurane showed that sevoflurane inhibited the proliferation, migration and invasion of ovarian cancer cells and induced apoptosis in a dose-dependent manner. Blocking the MAPK pathway can reduce the inhibitory effect of sevoflurane on ovarian cancer cells (Kang and Wang, 2019). Zhang et al. found that sevoflurane not only inhibited cell viability and colony formation ability of ovarian cancer cells, but also downregulated Cyclin D1/CDK4 to arrest the cell cycle in G0/G1 phase and induced apoptosis to play an anti-growth role. In addition, sevoflurane reduced the migration and invasion ability of ovarian cancer cells (Zhang et al., 2019).

However, in recent years, it has also been found that sevoflurane has a pro-tumor effect on ovarian cancer cells. The TWIK-related acid sensitive K^+^ channel-3 (TASK-3) is expressed in epithelial ovarian cancer, and it is involved in the progression of cancer by regulating the apoptosis and proliferation of ovarian cancer cells, so it is regarded as a new prognostic marker (Innamaa et al., 2013). Sevoflurane, as a TASK-3 channel activator, can promote the growth and metastasis, however, propofol can inhibit the migration and invasion of ovarian cancer cells (Hu et al., 2024). This is consistent with previous study showing that propofol inhibits glucose metabolism in ovarian cancer cells and plays an anti-cancer role, while sevoflurane has the opposite effect, which promotes the viability, proliferation, migration and invasion of ovarian cancer cells (Cong et al., 2022). An article examining the difference in long-term survival between sevoflurane and propofol in patients undergoing surgery for gynecologic cancers reported that recurrence-free survival (RFS), cancer-specific survival (CSS), and overall survival (OS) were worse in patients who received sevoflurane anesthesia, especially in those with ovarian cancer (Takeyama et al., 2021). However, in this study, the proportion of patients with clinical stage III/IV cancer was higher in the sevoflurane group; therefore, further studies are needed to determine the optimal anesthesia regimen for patients with gynecologic oncology to improve long-term survival.

At present, sevoflurane has both cancer-promoting and anti-cancer effects in cervical and ovarian cancer. However, the majority of evidence remains limited to preclinical animal models and *in vitro* studies. While *in vitro* models provide simplified experimental conditions, *in vivo* systems involve complex biological variables—such as the previously described immune modulation and metabolic reprogramming—that critically influence cancer cell metastasis. Furthermore, expanding clinical research is warranted to address existing knowledge gaps. Specifically, comparative clinical trials evaluating sevoflurane monotherapy versus its combination with other anesthetic agents (e.g., propofol, dexmedetomidine, and sufentanil) could clarify whether polypharmacological strategies mitigate potential limitations of sevoflurane in gynecologic cancer.

#### 3.2.2 Isoflurane

The main mechanism by which anesthesia affects tumors is immunomodulatory. In mice with normal immune function, isoflurane accelerates tumor growth and reduces the infiltration of lymphocytes and monocytes. Therefore, isoflurane promotes cancer progression by disrupting immune function (Abrahams et al., 2023). However, it has also been confirmed that isoflurane can increase the expression of pro-apoptotic genes, reduce the expression of anti-apoptotic genes, and inhibit the growth, migration and invasion of liver cancer cells (Hu et al., 2018). Therefore, the role of isoflurane in tumors is currently controversial, and its effects in gynecologic tumors are still unclear.

##### 3.2.2.1 Cervical cancer

The mammalian target of rapamycin (mTOR) is a key regulator of the immune response, which regulates the differentiation and function of adaptive and innate immune cells. Activation of the AMPK/mTOR signaling pathway can activate autophagy, which is one of the mechanisms of cell death in cervical cancer (Mafi et al., 2021; Yang et al., 2020). Isoflurane inhibits the viability of cervical cancer cells, induces apoptosis, enhances oxidative stress, and induces autophagy by activating AMPK/mTOR pathway. These effects have been confirmed *in vitro* and *in vivo*, indicating that isoflurane has anti-cancer effects on cervical cancer (Wei et al., 2021). However, isoflurane promotes the proliferation of squamous cervical cancer cells and increases the expression of histone deacetylase 6 (HDAC6) by activating AKT/mTOR signaling pathway (Zhang et al., 2021b). The effects of isoflurane on cervical cancer have been completely different, which may be related to the different mTOR upstream signaling molecules affected by isoflurane. In addition, different cervical cancer cells have their own biological characteristics, and experimental conditions also affect the experimental results. Compared with the former, the latter research lacks *in vivo* experimental models to further verify the *in vitro* results.

##### 3.2.2.2 Ovarian cancer

Isoflurane increased the expression of insulin-like growth factor (IGF)-1, IGF-1R, VEGF, MMP2 and MMP9 in ovarian cancer cells, enhanced angiogenesis ability, and significantly increased cell migration, which was the first time that isoflurane was found to increase the malignant potential of ovarian cancer cells through IGF1/HIF signaling pathway *in vitro* (Luo et al., 2015). Isoflurane can also regulate ovarian cancer cell metabolism, stimulate ovarian cancer cells glucose uptake, lactic acid and extracellular acidification, this has to do with isoflurane induced miR-21 expression significantly correlated (Guo et al., 2017). Volatile anesthetics altered the expression of most metastasis-related genes, with significant increases in MMP-11, CXCR2, VEGF-A, and TGF-β expression, with the greatest effect of desflurane and the least effect of isoflurane. Clinically relevant concentrations of volatile anesthetics increased the metastatic potential of ovarian cancer. In addition, sevoflurane and desflurane can also enhance the proliferation and the malignancy of ovarian cancer cells by inhibiting miRNA (Ishikawa et al., 2021; Iwasaki et al., 2016). At present, the research on isoflurane in ovarian cancer is few and in an adverse state. Exploring more effects of isoflurane on ovarian cancer and its related mechanisms can provide more and more comprehensive theoretical basis for clinical anesthesia.

Although the effects of most volatile anesthetics on gynecological tumors have conflicting results, the current research is basically based on animal studies, and clinical studies are still lacking. Therefore, the long-term effects of volatile anesthetics on patients with gynecological tumors need to be discussed. In addition, at the time of surgery, anesthesia can not only rely on a single volatile anesthetic to meet the needs of surgery, need to be completed by other intravenous drugs. Perhaps when a variety of drugs are used together, the effect of volatile anesthetics on gynecological cancer will turn from disadvantage to advantage.

### 3.3 Local anesthetics

#### 3.3.1 Lidocaine

Lidocaine is an amide local anesthetic, which is one of the most widely studied and used local anesthetics. Lidocaine can regulate immune and inflammatory responses, reduce surgical pain, reduce the survival ability of most tumor cells, and also have an effect on tumor cell migration and invasion, and also inhibits tumor cell metabolism to play an anti-cancer role. In addition, lidocaine combined with other general anesthetic drugs can reduce the use of the latter, and therefore, lidocaine has shown a more beneficial side in cancer (Karniel and Beitner, 2000; Liu et al., 2020; Zhang C. et al., 2021).

##### 3.3.1.1 Cervical cancer

A 2023 study reported that intravenous lidocaine before surgery increased disease-free survival (DFS) and OS in cancer patients (Badwe et al., 2023). Ki-67/MIB-1 protein, a marker of cell proliferation, is one of the tumor markers. A meta-analysis showed that cervical cancer patients with high Ki-67/MIB1 expression had shorter OS, suggesting that Ki-67/MIB-1 has prognostic value for OS in cervical cancer patients (Pan et al., 2015). Lidocaine can induce Ki-67 degradation in the nucleus of cervical cancer cells, thereby inhibiting the growth of cancer cells, and arrest cervical cancer cells in G1 phase and delay their entry into S phase (Haraguchi-Suzuki et al., 2022).

MiR-421 reduces glucose consumption and lactate production, and is a negative regulator of aerobic glycolysis in cancer cells (Meng et al., 2016). Study on the molecular mechanism between lidocaine and cervical cancer cells found that lidocaine inhibits tumor growth by inhibiting cervical cancer cell viability and inducing apoptosis by regulating the lncRNA-MEG3/miR-421/BTG1 pathway, which provides a new direction for the clinical treatment of cervical cancer (Zhu and Han, 2019). Lidocaine could induce lymphocyte proliferation and maintain Th1/Th2 cell balance, which could protect cell-mediated immunity (CMI) and reduce the risk of cancer metastasis in cervical cancer patients (Wang et al., 2015). From the above results, it is reasonable to conclude that lidocaine has a beneficial effect on the prognosis of cervical cancer cells.

##### 3.3.1.2 Ovarian cancer

Lidocaine can reduce the viability and inhibit the invasion and migration of ovarian cancer cells. The anti-cancer effect of lidocaine may be related to the reduction of calcium influx and the reduction of TRPV6 expression (Jiang et al., 2016). Subsequently, the anti-cancer mechanism of lidocaine in ovarian cancer was explored, and it was found that lidocaine promoted iron ptosis through MiR-382-5p/SLC7A11 axis, thereby inhibiting the cell viability and proliferation of ovarian cancer cells, inducing apoptosis, and weakening invasion and migration ability (Sun D. et al., 2021). Lidocaine can inhibit EMT of ovarian cancer cells through the Wnt/β-catenin pathway, and can inhibit the malignant progression of ovarian cancer cells (Sun M. et al., 2021). Lidocaine as a TASK-3 channel inhibitor can inhibit the growth and metastasis of ovarian cancer cells *in vivo* and *in vitro*, and reduce lung metastasis *in vivo* (Hu et al., 2024). It can be seen that the advantages of lidocaine in ovarian cancer are outstanding. In addition, lidocaine enhances the sensitivity of ovarian cancer cells to cisplatin (Liu et al., 2021), and perhaps lidocaine can be used as an adjuvant therapy for ovarian cancer patients in the future.

It can be seen from the above that lidocaine can play an anti-cancer role in ovarian cancer through multiple signaling pathways, but its role in clinical practice still needs further study. In a clinical study, researchers injected lidocaine 1.5 mg/kg at induction of general anesthesia, and continued infusion of 2 mg/(kg∙h) during surgery until the end of surgery, they found that lidocaine prolonged OS and DFS of ovarian cancer patients (Zhang et al., 2021b).

##### 3.3.1.3 Endometrial cancer

Autophagy regulates innate and adaptive immune responses, including influencing the inflammatory environment and the development, survival, and effector functions of various immune cells. Autophagy has a dual impact on cancer biology, which may promote the development of endometrial cancer by enabling cell survival in adverse environments (Devis-Jauregui et al., 2021; Yamazaki et al., 2021). However, lidocaine has been found to inhibit the viability, proliferation and migration of endometrial cancer cells, and promote cell apoptosis by inducing autophagy (Long et al., 2022). Therefore, lidocaine has an anti-cancer effect on endometrial cancer and may be a potential therapeutic agent for endometrial cancer. However, since this is only an *in vitro* study, further confirmation by *in vivo* studies and clinical studies are lacking, more studies are needed to explore the effect of lidocaine on endometrial cancer.

The effect of lidocaine on gynecological cancer is mainly reflected in anti-cancer effect, which can inhibit the proliferation and migration of cancer cells and promote the apoptosis of cancer cells. Local application of lidocaine or intravenous injection can be considered. Intravenous injection can not only reduce the cough during tracheal intubation, but also prevent ventricular arrhythmia.

#### 3.3.2 Ropivacaine

Ropivacaine is a long-acting amide local anesthetic, which was used in clinic in 1996, and it is safer than bupivacaine, so it is widely used in clinic. A large number of studies have found that ropivacaine inhibits cancer cell metabolism and proliferation through a variety of signaling pathways, such as Ras, RhoA and Wnt/β-catenin signaling pathway. In addition, ropivacaine can also inhibit the invasion and migration of cancer cells, and shorten the recovery time for cancer patients (Owen and Dean, 2000; Xu et al., 2022). Therefore, it is feasible to explore the mechanism of action of ropivacaine in gynecologic cancer.

##### 3.3.2.1 Cervical cancer

Targeted inhibition of signal transducer and activator of transcription 3 (STAT3) may play an anti-cancer role by improving the tumor immune microenvironment and changing the metabolism of cancer cells (Li Y. et al., 2022). STAT3 is highly expressed in cervical cancer, and the expression of STAT3 protein is positively correlated with TNM stage of cervical cancer, depth of invasion and lymph node metastasis (Shi et al., 2021; Wu L. et al., 2022). Ropivacaine can inhibit the cell cycle process and induce cell apoptosis of cervical cancer cells by inhibiting miR613/MEG2/pSTAT3 pathway, thus inhibit the growth of cervical cancer cells (Chen et al., 2020). The effect of ropivacaine on cervical cancer cells was observed by loading ropivacaine into tumor active targeting liposomes. It was found that ropivacaine could disrupt autophagic degradation of cancer cells, promote cell death caused by nutrient deprivation, and inhibit tumor growth. In addition, ropivacaine could also reduce cancer pain (Zhang et al., 2020).

##### 3.3.2.2 Ovarian cancer

Studies have found that ropivacaine, an inhibitor of NaV1.5, can attenuate the invasion ability of colon cancer cells (Baptista-Hon et al., 2014). As previously reported, lidocaine inhibits ovarian cancer cell growth, migration, and invasion by blocking Nav1.5 and its downstream pathway (Liu et al., 2021). It is reasonable to infer that ropivacaine exerts its anti-cancer effect in ovarian cancer cells through Nav1.5. In addition, local injection of ropivacaine can shorten the interval between the start of chemotherapy after surgery in ovarian cancer patients, which may have a beneficial effect on the prognosis of ovarian cancer patients (Hayden et al., 2020). However, in a recent phase III trial, it was found that local injection of ropivacaine did not shorten the time to return to intended oncology therapy (RIOT) after ovarian cancer surgery (Hasselgren et al., 2024).

The new use of old drugs is a hot spot, which can let us know more about the pharmacological effects of drugs. Secondly, it can provide anesthesiologists with better anesthesia programs. Lidocaine and ropivacaine have shown anticancer effects in gynecological cancer, and we believe that there may be more clinical studies to explore their effects on gynecological cancer in the future.

### 3.4 Other drugs

#### 3.4.1 Opioids

Opioids are considered as immune modulators. Opioids can not only directly affect immune cells, such as neutrophils, macrophages and NK cells, but also affect immune function through the HPA axis. In addition, opioids can also regulate sympathetic nerve activity to affect immune function. Therefore, the use of opioids in cancer patients needs to weigh the pros and cons. Pain is a common symptom in patients with advanced cancer. Opioids are dominant in patients with cancer pain, but their immunosuppressive effect brings challenges to opioids (Al-Hashimi et al., 2013; Bradley and Boland, 2023; Li et al., 2024). Opioids can also regulate cancer cell metabolism, such as glycolysis, creatine metabolism and glutamine metabolism (Tarazi and Maynes, 2023). Understanding the mechanism and changes of metabolic pathways in gynecological cancer will help to better discover the role of opioids in gynecological cancer.

##### 3.4.1.1 Cervical cancer

Epidermal growth factor (EGF) signaling is the first step in activating glycolysis, and inhibition of EGF receptor (EGFR) inhibits cancer cell proliferation and tumor growth. In addition, knockdown of EGFR reduces levels of glycolytic metabolites, glucose uptake and lactate production (Kim et al., 2018; Lim et al., 2016). EGFR is highly expressed in cervical cancer tissues and cells. Knockdown of EGFR can inhibit the proliferation, migration and invasion, and promote apoptosis of cervical cancer cells (Yang et al., 2023). Morphine promotes cell proliferation through opioid receptor-dependent activation of EGFR and stimulates cell migration through RhoA-independent activation, but it has no significant effect on cervical cancer cell apoptosis. In addition, morphine can reduce the efficacy of chemotherapy drugs on cervical cancer (Yu et al., 2022). Study has found that fentanyl is safe and effective in the use of cervical cancer patients, which can reduce pain and improve the quality of life of cervical cancer patients for a long time (Thanthong et al., 2017). The use of opioids is essential after the onset of pain symptoms in cervical cancer patients; therefore, more research is needed to explore the optimal choice of opioid use in cervical cancer patients.

##### 3.4.1.2 Ovarian cancer

EGFR is not only abnormally expressed in cervical cancer, but also overexpressed in ovarian cancer, and downregulation of EGFR expression inhibits ovarian cancer cell proliferation (Dou et al., 2021). Fentanyl can specifically activate EGFR and its downstream MEK/ERK and PI3K/Akt signaling pathways, thereby promoting the proliferation and migration of ovarian cancer cells, but has no significant effect on cell apoptosis, and can reduce the death of ovarian cancer cells when combined with chemotherapy drugs (Xiao et al., 2022). A small retrospective study found that ovarian cancer patients treated with general anesthesia and fentanyl had a worse long-term prognosis than those treated with epidural anesthesia and analgesia (Lin et al., 2011), which is consistent with previous studies showing that local anesthetics lidocaine and ropivacaine have shown anti-cancer effects. Sufentanil and fentanyl are commonly used analgesics for patients with cancer pain. Both of them mainly act on mu-opioid receptors (MOR), which is involved in tumor proliferation, invasion, metastasis and angiogenesis, and may be a new target for cancer treatment (Zhang, et al., 2021c). A retrospective cohort study found that patients with high MOR expression required more sufentanil during surgery and had a higher risk of peripheral nerve invasion. However, MOR expression level was not associated with OS and DFS (Zhang et al., 2022a).

Opioids are at a disadvantage in gynecological tumors, mainly showing a cancer-promoting effect, but the use of opioids in patients with cancer pain is essential. Because there is a lack of research on opioids and gynecological tumors, more studies are needed to explore the relationship between them.

#### 3.4.2 Dexamethasone (dex)

Dexamethasone (Dex) is a kind of glucocorticoid steroid, which is commonly used as an adjuvant drug in anesthesia. Dex is regulated by the HPA axis and participates in a variety of body activities, such as anti-inflammatory effect, immunosuppression, reduction of stress response, antiemetic, and enhanced analgesic effect when combined with analgesics (Chu et al., 2014). In addition, Dex can also inhibit glycolysis and induce autophagy, and high-dose Dex can reduce the expression of glycolysis-associated genes and lipid-associated genes, thereby inhibiting tumor progression (Gu et al., 2015; Xu L. et al., 2020).

##### 3.4.2.1 Cervical cancer

The coxsackie and adenovirus receptor (CAR) is a transmembrane glycoprotein that inhibits cervical cancer cell migration (Brüning and Runnebaum, 2004). Dex downregulates CAR expression, resulting in reduced adenoviral gene transfer to human cancer cells, and the use of Dex should be carefully considered in the treatment of cervical cancer with adenovirus vectors (Brüning and Runnebaum, 2003). Early studies found that Dex inhibited cervical cancer cell apoptosis, which could be reversed by the use of hormone antagonists (Kamradt et al., 2000). However, a subsequent study of ursolic acid (a chemically similar substance to Dex) showed that ursolic acid inhibited the proliferation of HPV-associated cervical cancer cells and induced apoptosis, but Dex did not affect cervical cancer cell proliferation (Yim et al., 2006). In a study of human cervical cancer specimens, glucocorticoid receptor (GR) expression was found to be increased in cervical cancer tissues, and OS and progression-free survival were better with high GR expression (Kost et al., 2019). Dex is a GR agonist.

##### 3.4.2.2 Ovarian cancer

As early as more than a dozen years ago, Dex was found to change the gene expression of ovarian tumors and affect the efficacy of chemotherapy in ovarian cancer patients (Melhem et al., 2009). Subsequently, Dex was found to affect chemotherapy-induced apoptosis by promoting ECM adhesion in human ovarian cancer cells (Chen et al., 2010). However, Dex was not associated with postoperative ovarian cancer recurrence (De Oliveira et al., 2014). Targeting ROR1 expression can improve the efficacy of chemotherapeutics (such as taxanes) and anti-apoptotic compounds (such as SMAC mimics) in ovarian cancer. Dex can increase ROR1 expression and enhance the sensitivity of AKT/PI3K targeted kinase inhibitors, and reduce the cytotoxic effects of taxanes and SMAC mimetics (Karvonen et al., 2020). A retrospective cohort study showed that early administration of Dex was an effective adjuvant therapy for ovarian cancer patients with high short-term mortality after the onset of symptoms associated with malignant small bowel obstruction (MSBO) (Jones et al., 2022).

##### 3.4.2.3 Endometrial cancer

In 1999, 11b-HSD2 was found to be specifically expressed in human endometrial adenocarcinoma cells and Dex increased 11b-HSD2 activity, suggesting that the human endometrium is a target tissue for glucocorticoids (Darnel et al., 1999). Dex can inhibit the growth and invasion of endometrial cancer cells by inducing the expression of anti-proliferative factor c-fos relating transcription factor-2 (USF-2) and down-regulating the expression of cell adhesion molecule (n-cadherin) (Davies et al., 2006). However, it has also been found that GR is associated with poor prognosis of endometrial cancer, GR can increase the invasiveness of endometrial cancer cells with high expression of estrogen receptor (ER), and Dex and estradiol (E2) can promote the growth of endometrial cancer cells after co-treatment (Vahrenkamp et al., 2018).

Dex is a commonly used adjuvant drug for cancer patients, but its effect on the recurrence and metastasis of gynecological tumors is still unclear. More studies are needed to explore the effect of Dex on gynecological tumors and its related mechanisms.

## 4 Summary

Combined with the above complaints, intravenous anesthetics (propofol and dexmedetomidine) and local anesthetics (lidocaine and ropivacaine) have mainly shown anti-cancer effects in gynecological tumors. However, not all anesthetic agents are uniformly favorable in gynecological tumors. The majority of volatile anesthetics exhibit dual effects including both cancer-promoting and anti-cancer effects, though current evidence exclusively indicates cancer-promoting effects of isoflurane in ovarian cancer. Opioids mainly show cancer-promoting effects, including morphine on cervical cancer and fentanyl and sufentanil on ovarian cancer. The impact of dexamethasone (Dex) on gynecological malignancies remains ambiguous, with reported anti-cancer effects in cervical cancer but conflicting reports regarding its role in endometrial carcinomas, where both cancer-promoting and anti-cancer effects have been documented. However, existing research on anesthetic agents and gynecologic cancers predominantly relies on preclinical animal models, and there is a lack of clinical experiments to explore their role in practical clinical work. Therefore, more studies are needed to explore the impact of anesthetics on gynecologic cancer. Finally, we summarize the effects of anesthetics on gynecological cancer and its related mechanisms *in vitro* (Table 1).

**TABLE 1:** Effect of anesthetics on gynecological tumors.

Anaesthetic	Type of cancer	Cell resource	Effect	Mechanism	References
Propofol	Cervical cancer	HeLa	Promotes autophagosome accumulation	Induction of ER stress and regulation of AMPK/mTOR signaling	Chen et al. (2018)
		Caski and SiHa cell	Restrains the growth and invasion capacity of cervical carcinoma cells	Inhibits MIR155HG	Du et al. (2021)
		Hela, Caski, C-33A and SiHa	Inhibits tumor size, cell viability and promotes apoptosis	Inhibits mTOR/p70S6K pathway mediated by HOTAIR and HOTAIR/miR-129-5p/RPL14 axis	Sun et al. (2021c); Zhang et al. (2015)
	Ovarian cancer	ES-2, IOSE-80, A2780, SKOV3, 293 T and OVCAR-3	Inhibits cell proliferation, migration, invasion and induces apoptosis	Inhibits the activation of MEK/ERK signaling and the JAK2/STAT3 signal pathway, upregulates miR-9 expression and inhibits NF-kB activation and its downstream MMP-9 expression	Huang et al. (2016); Lu et al. (2021); Shen et al. (2021)
		SKOV3 and OVCAR3	Suppresses colony formation, disrupts mitochondrial membrane potential, and induces apoptosis and autophagy	Inhibits the ovarian cancer cells either alone or in combination with chemotherapy agents	Sue et al. (2024)
	Endometrial cancer	Ishikawa	Inhibits cell proliferation, migration, and invasion but promotes apoptosis	Inhibits Sox4 expression via inactivation of Wnt/β-catenin signal pathway	Du et al. (2018)
Dexmedetomidine	Ovarian cancer	SK-OV-3	Promotes the recovery of NK cell activity and reduce TNF-α level	Suppresses the SSR and stress mediator release	Shin et al. (2021)
		SKOV3 and HO-8910	Inhibits cell proliferation, invasion and migration	Upregulates miR-185 expression and inactivates the SOX9/Wnt/β-catenin signaling pathway	Tian et al. (2021)
Sevoflurane	Cervical cancer	Siha and Hela	Promotes cell proliferation and migration, inhibits apoptosis	—	Xue et al. (2019)
		HeLa, SiHa and C-33A	Inhibits cell proliferation and migration	Inhibits Ras and RhoA GTPase activities	Ding et al. (2019)
	Ovarian cancer	SKOV3 and OVCAR3	Inhibits cell proliferation, migration, and invasion but promotes apoptosis	Inhibits JNK and p38 MAPK signaling pathway and the expression of STC1	Kang and Wang (2019); Zhang et al. (2019)
		SKOV3	Promotes cell proliferation and migration	Upregulates the Erk1/2 pathway and activates TASK-3 channel	Cong et al. (2022); Hu et al. (2024)
Isoflurane	Cervical cancer	Hela	Inhibits cell viability, triggers oxidative stress, induces apoptosis and autophagy	Activates the AMPK/mTOR pathway	Wei et al. (2021)
		SiHa and Caski	Enhances cell proliferation	Activates the AKT/mTOR pathway	Zhang et al. (2021d)
	Ovarian cancer	SKOV3 and TOV21G	Increases cell proliferation, angiogenesis, upregulates glucose uptake and lactate production	Upregulates miR-21 and AKT phosphorylation	Guo et al. (2017), Luo et al. (2015)
Lidocaine	Cervical cancer	Hela	Increases cell population in the G0/G1 phase	Regulates the expression and distribution of Ki-67	Haraguchi-Suzuki et al. (2022)
		Hela	Inhibits cell proliferation and induces apoptosis	Modulates the lncRNA-MEG3/miR-421/BTG1 pathway	Zhu and Han (2019)
	Ovarian cancer	ES-2, SKOV3	Inhibits cell migration and invasion	Reduces rate of calcium influx and downregulates TRPV6 expression, promotes ferroptosis by miR-382-5p/SLC7A11 axis	Jiang et al. (2016); Sun D. et al. (2021)
	Endometrial cancer	RL95-2	Inhibits cell viability, proliferation, migration and induces apoptosis	—	Long et al. (2022)
Ropivacaine	Cervical cancer	Siha and Caski	Inhibits cell cycle progression and promotes apoptosis	Inhibits the miR613/MEG2/pSTAT3 axis	Chen et al. (2020)
Opioids	Cervical cancer	C-33A and CaSki	Promotes proliferation and migration	Activates EGFR-mediated and RhoA-mediated signaling pathways	Yu et al. (2022)
	Ovarian cancer	SW626, SKOV3 and TOV21G	Promotes proliferation and migration	Activates EGFR and its-mediated downstream pathways	Xiao et al. (2022)
Dexamethasone	Ovarian cancer	SKOV3 and HO-8910	Promotes ECM adhesion	Enhances the protein levels of members of integrin β1 subfamily and TGF-β1-signaling pathway	Chen et al. (2010)
	Endometrial cancer	Ishikawa H	Inhibits cell growth and invasion	Induces USF-2 and downregulates N-cadherin	Davies et al. (2006)

Can we speculate that the combination of spinal anesthesia and general anesthesia is more suitable for gynecological tumor patients? The local anesthetics used in spinal anesthesia mainly play an anti-cancer effect on gynecological tumor, and at the same time, it can also reduce the dosage of general anesthetics and other adjuvant drugs to reduce the impact on cancer cells. However, a recent large study found that paravertebral block combined with propofol anesthesia did not reduce cancer recurrence compared with sevoflurane and opioid anesthesia (Sessler et al., 2019). In addition, patients undergoing gynecological cancer surgery are generally older on average, and the neuraxial anesthesia technique has higher requirements for anesthesiologists, which brings higher challenges to anesthesia. For gynecologic oncology patients, a combined neuraxial-general anesthesia approach is recommended as the preferred anesthetic strategy. In cases where neuraxial anesthesia is contraindicated or technically challenging, the current ultrasound-guided nerve block combined general anesthesia can be chosen.
